# The Utility of a Community-Based Knee Ultrasound in Detecting Meniscal Tears: A Retrospective Analysis in Comparison with MRI

**DOI:** 10.3390/healthcare12202051

**Published:** 2024-10-16

**Authors:** Fatima Awan, Prosanta Mondal, Johannes M. van der Merwe, Nicholas Vassos, Haron Obaid

**Affiliations:** 1 College of Medicine, University of Saskatchewan, Saskatoon, SK S7N 5E5, Canada; fza921@mail.usask.ca; 2 Clinical Research Support Unit, College of Medicine, University of Saskatchewan, Saskatoon, SK S7N 5A9, Canada; prosanta.mondal@usask.ca; 3 Section of Orthopedic Surgery, Department of Surgery, College of Medicine, University of Saskatchewan, Saskatoon, SK S7K 0M5, Canada; jov777@mail.usask.ca; 4 Department of Medical Imaging, College of Medicine, University of Saskatchewan, Saskatoon, SK S7N 5A9, Canada; nav353@mail.usask.ca

**Keywords:** meniscus, tibia, knee injuries, diagnostic imaging, prevalence, predictive value of tests

## Abstract

Background/Objectives: MRI is the gold standard for detecting meniscal tears; however, ultrasound may readily detect meniscal changes, obviating the need for MRI. We aim to (1) determine ultrasound sensitivity, specificity, positive predictive value (PPV), negative predictive value (NPV), and accuracy in detecting meniscal changes, and (2) describe characteristic meniscal changes in US and their prevalence. Methods: A retrospective analysis of knee ultrasound scans for the presence of medial and lateral meniscal tears was conducted. Meniscal changes were characterized into five US appearances (cleft, diminutive, cyst, displaced fragment, and extrusion) by the consensus of two musculoskeletal radiologists. Ultrasound findings were then compared to MRI results. Results: In total, 249 patients were included. Ultrasound sensitivity, specificity, PPV, NPV, and accuracy for medial meniscal tears were 79%, 97.3%, 95.3%, 86.6%, and 90%, respectively, and for lateral meniscal tears the ultrasound sensitivity, specificity, PPV, NPV, and accuracy were 63%, 99.5%, 96%, 93%, and 93.6%, respectively. The false negative and false positive rates for medial meniscal tears were 13.4% and 4.7%, respectively, and for the lateral meniscus, the false negative and false positive rates were 6.7% and 3.8%, respectively. Meniscal clefts were the most prevalent appearance in the medial meniscus followed by extrusions. Meniscal extrusions were the most prevalent appearance in the lateral meniscus followed by clefts. Conclusions: Community-based US is highly accurate in the detection of meniscal tears when compared with MRI, making it a valuable diagnostic imaging tool for detecting meniscal tears in a community setting where accessibility to MRI is limited or if there are MRI contraindications.

## 1. Introduction

The meniscus represents a vital component of the knee and plays a major role in knee function. It represents a C-shaped fibrocartilaginous structure between the femur and tibia which helps with the biomechanics of the knee, especially with shock absorption and load transmission [1].

Injuries to the meniscus are relatively common in clinical practice, which makes diagnosing these injuries imperative to improving patients’ symptoms and quality of life [2]. Previous studies have shown a prevalence of 12% to 14% and an incidence of 60 cases per 100,000 people in the United States [3,4]. The prevalence of meniscal tears is high in individuals with osteoarthritis [5,6]. The timely diagnosis of tears is therefore necessary to reduce costs and provide effective treatment [7]. Of the estimated 850,000 cases of meniscal tears per year in the United States, meniscal surgery is performed in 10–20%, and is associated with high annual costs [8,9].

Magnetic Resonance Imaging (MRI) is regarded as the gold standard for detecting injuries to the meniscus [10,11]. While MRI has the strength to provide highly detailed images with superior contrast resolution, it has several limitations and pitfalls [12]. Its main limitations are its high costs and limited availability, primarily restricted to hospital settings. In addition, many patients are denied MRI examinations due to contraindications, such as cardiac pacemakers, claustrophobia, and periorbital foreign bodies [13,14]. To overcome these limitations, ultrasonography (US) has become a valuable modality due to its high spatial resolution, low-cost operation, and community-based availability outside hospital settings [15]. Another advantage to US is the ability to perform dynamic imaging, correlate this with patients’ symptoms, compare this with the contralateral side, and provide therapeutic interventions which can be performed in point-of-care settings [16]. Previous studies have shown variable results regarding US sensitivity, specificity, and accuracy in detecting meniscal tears [17,18]. We hypothesize that a community-based ultrasound can provide accurate detection of meniscal tears using characteristic appearances, which are parameniscal cysts, displaced meniscal fragments, diminutive meniscus, meniscal cleft, and meniscal extrusion.

## 2. Materials and Methods

### 2.1. Study Desing and Setting

A retrospective analysis was conducted between Jan 2024 and July 2024 to evaluate community-based knee ultrasounds for the presence of medial or lateral meniscus tears. Research Ethics Board approval was obtained from the Institutional Ethics Board (Bio 4489). Informed consent was waived by the Institutional Ethics Board.

### 2.2. Participants

The inclusion criteria for the study: patients were men or women between the ages of 18 and 55 years, and a US and MRI had been performed within three months of each other. All ultrasound scans were performed in the community and images were obtained by musculoskeletal-trained ultrasound technologists. All MRI scans were reported by musculoskeletal-trained radiologists. The exclusion criteria: any patient who did not meet the inclusion criteria, suboptimal MRI images, incomplete scans, and patients with history of prior knee surgery, infection, or inflammation.

### 2.3. Imaging Techniques and Analysis

All US images were obtained in a community setting using L12-5 Linear probe (12–17 MHz) Phillips IU22 machines (Philips, Amsterdam, The Netherlands) by dedicated musculoskeletal-trained sonographers. Consensus reads on the ultrasound findings (presence or absence of meniscal tears) were performed by two fellowship-trained musculoskeletal radiologists [19]. The two radiologists were blinded to the MRI findings. The meniscal changes were characterized into five US criteria: meniscal cleft, parameniscal cyst, diminutive menisci, displaced meniscal fragment, and meniscal extrusion.

The MRI was obtained on a 3 Tesla Skyra Siemens MRI scanner (MAGNETO Skyra; Siemens Healthcare, Erlangen, Germany). MRI protocol included high-resolution 3D coronal proton density (PD) Sampling Perfection with Application optimized Contrasts using different flip angle Evolution (SPACE) (TR/TE = 1200/27, slice = 0.75 mm, field of view = 160 mm, acquisition matrix = 380 × 304, phase encode direction right–left, receiver bandwidth = 385 Hz/pixel, echo train length = 23, reconstructed resolution 0.5 × 0.5 × 0.8 mm); coronal PD turbo spin echo (TSE) with fat saturation (FS) (TR/TE = 2500/33, slice = 3 mm, field of view = 150 mm, acquisition matrix = 320 × 256, phase encode direction right–left, receiver bandwidth = 248 Hz/pixel, echo train length = 8, reconstructed resolution 0.5 × 0.5 × 3 mm); axial PD TSE FS (TR/TE = 3500/33, slice = 3 mm, field of view = 160 mm, acquisition matrix = 320 × 240, phase encode direction right–-left, receiver bandwidth = 244 Hz/pixel, echo train length = 8, reconstructed resolution = 0.5 × 0.5 × 3 mm); sagittal PD TSE FS (TR/TE = 4310/33, slice = 3 mm, field of view = 160 mm, acquisition matrix = 320 × 272, phase encode direction head–foot, receiver bandwidth = 248 Hz/pixel, echo train length = 8, reconstructed resolution = 0.5 × 0.5 × 3 mm); and sagittal T1-weighted TSE (TR/TE = 971/12, slice = 3 mm, field of view = 160 mm, acquisition matrix = 384 × 307, phase encode direction anterior–posterior, receiver bandwidth = 150 Hz/pixel, echo train length = 91, reconstructed resolution = 0.4 × 0.4 × 3 mm).

### 2.4. Statistical Analysis

Analysis was carried out using packaged statistical software (IBM SPSS Statistics for Windows, Version 20.0. Armonk, NY, USA: IBM Corp). The menisci were assessed for tears versus no tears. The frequences of meniscal changes (cleft, parameniscal cyst, diminutive meniscus, displaced meniscal fragment, and meniscal extrusion) were analyzed. The meniscal tear frequencies and percentages in US and MRI were compared to assess sensitivity, specificity, negative predictive value, positive predictive value, and accuracy of ultrasound. The false negative and false positives rates for the ultrasound detection of meniscal tears were calculated. Sample size calculation was performed by the Clinical Statistical Unit at our institute.

### 2.5. US Criteria

**Figure 1:**
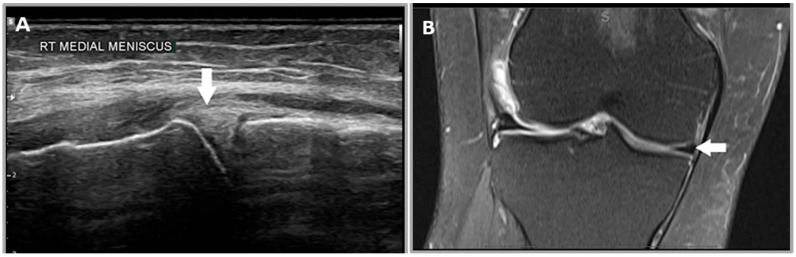
A 30-year-old male patient’s right knee. (**A**) Illustrates a normal meniscus in US which has a normal hyperechoic triangular appearance (white arrow). (**B**) A coronal proton density MRI image of the medial meniscus, which has a normal hypointense triangular appearance (white arrow).

A normal meniscus in US is defined as a triangular hyperechoic structure of normal size without a focal defect, parameniscal cyst, extrusion, or displaced fragment (Figure 1) [15,20].

#### 2.5.1. Criterion 1—Meniscal Cleft

**Figure 2:**
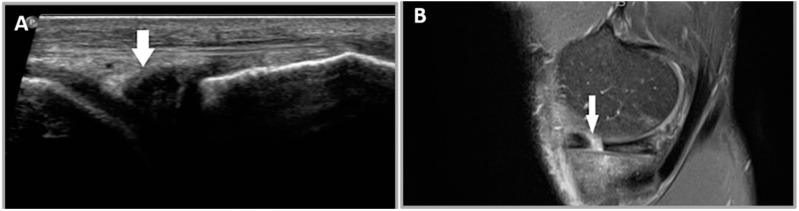
A 28-year-old female patient who presented with a history of left knee injury and medial sided knee pain. (**A**) A meniscal cleft is seen as a hypoechoic defect in the body of the medial meniscus (white arrow). (**B**) A sagittal proton density MRI image demonstrates a hyperintense defect in the body of the medial meniscus in keeping with a radial tear (white arrow).

A meniscal cleft in US is defined as a focal hypoechoic defect which corresponds to a hyperintense signal defect in MRI (Figure 2) [15,21].

#### 2.5.2. Criterion 2—Parameniscal Cyst

**Figure 3:**
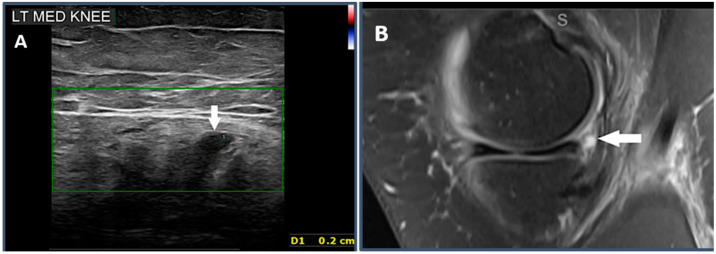
A 37-year-old male patient who presented with a history of medial sided knee pain. (**A**) A parameniscal cyst is seen as an anechoic structure adjacent to the posteromedial corner of the medial meniscus, measuring 2 mm (white arrow). (**B**) A sagittal proton density MRI image showing a focal fluid intensity area adjacent to the posteromedial corner of the medial meniscus (white arrow).

A parameniscal cyst is an indirect sign of a meniscal tear. This is seen as a focal anechoic structure adjacent to the meniscus which appears as a fluid signal intensity area in MRI (Figure 3) [22,23].

#### 2.5.3. Criterion 3—Diminutive Meniscus 

**Figure 4:**
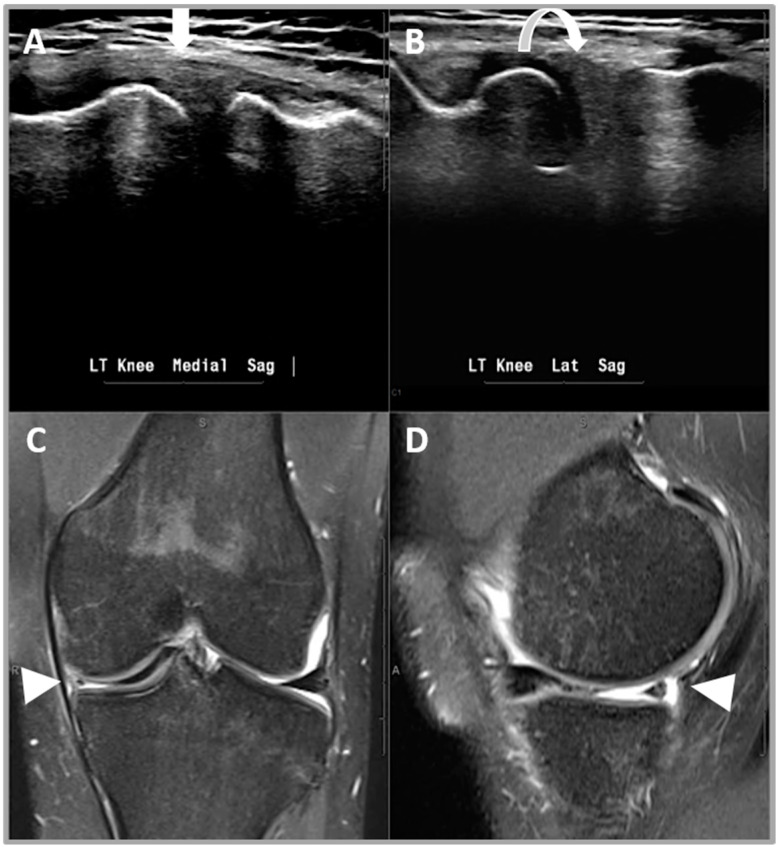
A 41-year-old male patient who presented with left knee joint locking and medial sided knee pain. (**A**,**B**) US appearances of the diminutive left medial meniscus (white arrow) when compared with the left lateral meniscus (curved arrow). (**C**) A coronal proton density image and (**D**) a sagittal proton density image demonstrating a diminutive medial meniscus due to a longitudinal vertical tear with a bucket handle fragment in the intercondylar notch (arrow heads).

A diminutive body is characterized by the meniscus size noted to be smaller than usual or when compared with the contralateral side (Figure 4) [20].

#### 2.5.4. Criterion 4—Displaced Meniscal Fragment

**Figure 5:**
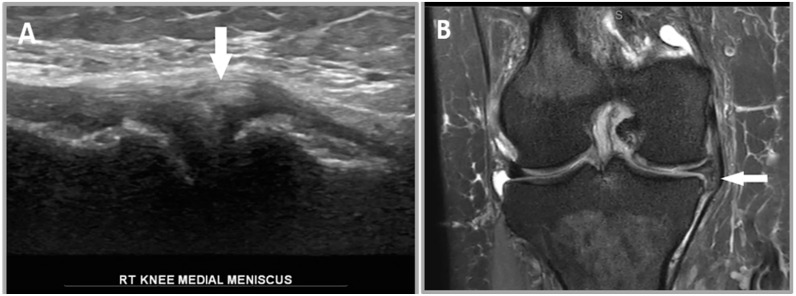
A 35-year-old female patient who presented with right knee locking and medial sided knee joint line pain. (**A**) A US image demonstrating extra meniscal tissue adjacent to the body of the medial meniscus (white arrow). (**B**) A coronal proton density image demonstrating a medial meniscal tear with a medially displaced meniscal fragment in the inferior gutter (white arrow).

A displaced meniscal fragment is defined as extra meniscal tissue that is not confined to the normal anatomic location (Figure 5) [24,25].

#### 2.5.5. Criterion 5—Extruded Meniscus

**Figure 6:**
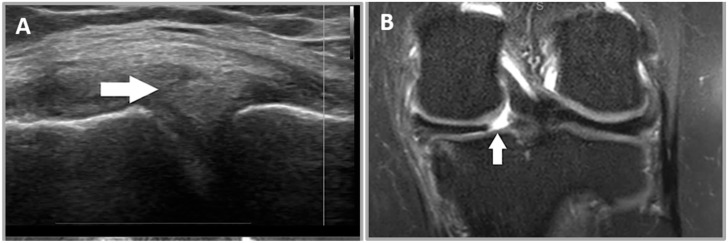
A 45-year-old female patient who presented with left knee joint twisting injury and medial sided knee pain. (**A**) US demonstrated extruded medial meniscus, which protrudes beyond the medial tibiofemoral joint line. (**B**) A coronal proton density MRI image demonstrating a full thickness radial tear of the posterior root attachment (white arrow) with medial meniscal extrusion.

An extruded meniscus is defined as the ≥3 mm radial external displacement of the meniscus from the tibiofemoral joint line (Figure 6) [26,27].

## 3. Results

A total of 600 cases were reviewed, and 249 patients (F = 113, M = 136) met our criteria. All knee ultrasound scans were performed in the community for clinical indications such as pain, locking, swelling, stiffness, and giving way. Patient ages ranged between 18 and 55. The median age was 38 years, and the average age was 37.8 years.

Of the menisci assessed, 121 were medial and 128 were lateral. All cases were assessed for the presence of a medial or lateral meniscal tears. The distribution of meniscal appearances in US is illustrated in Table 1.

### 3.1. Medial Meniscus

**Figure 7:**
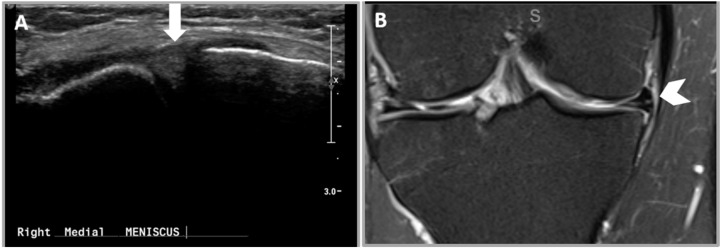
False negative medial meniscal tear in US. (**A**) US image demonstrating normal medial meniscus (white arrow). (**B**) A coronal proton density MRI image of the right knee demonstrating a horizontal tear of the medial meniscal body (white chevron).

**Figure 8:**
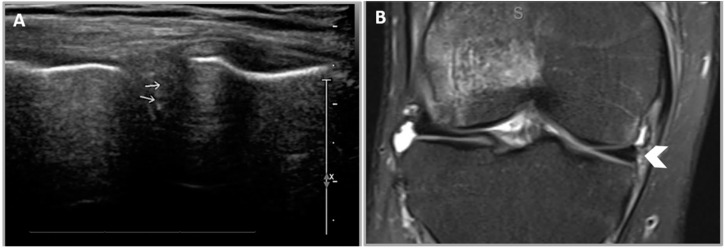
False-positive medial meniscal tear of the right knee in US. (**A**) US image of the right medial meniscus demonstrating small meniscal clefts (white arrows). (**B**) A coronal proton density MRI image demonstrating a normal medial meniscus (white chevron).

Using US imaging, 85 medial meniscal tears were identified (Table 2). An analysis of the MRI images showed the presence of 103 medial meniscal tears (Table 2). With regard to the medial meniscus, the sensitivity and specificity of US in comparison to MRI were 79% and 97.3%, respectively. The positive predictive value (PPV) and negative predictive value (NPV) were 95.3% and 86.6%, respectively. Overall, the US accuracy for the medial meniscus was 90%. The number of ultrasound false-negative and false-positive medial meniscal tears was 22 (13.4%) and 4 (4.7%), respectively (Figure 7 and Figure 8) (Table 3).

### 3.2. Lateral Meniscus

US imaging for the lateral meniscus showed 27 meniscal tears (Table 4), while the MRI showed 41 (Table 4). The sensitivity and specificity of the lateral meniscal tears were 63% and 99.5%, respectively. The positive and negative predictive values were 96% and 93%, respectively. The accuracy of US in detecting lateral meniscus tears was 93.6%. False-negative and false-positive lateral meniscal tears were 15 (6.7%) and 1 (3.8%), respectively (Table 5).

## 4. Discussion

The results of this study have shown that ultrasound sensitivity, specificity, PPV, NPV, and accuracy for medial meniscal changes were comparable to the upper limits of the reported ranges in the literature, with slightly higher values for specificity in our study (97.3% versus 96%). With regard to the lateral meniscal changes, the PPV, NPV, and accuracy were comparable to the upper limits of the reported ranges in the literature; however, the specificity was slightly higher than the literature (99.5% versus 98%), and the sensitivity was at the lower limits of the literature (63% versus 64%) [15,17,28,29]. The low ultrasound sensitivity for detecting lateral meniscal changes compared to the medial meniscal changes was previously reported in the literature [23,28]. A study by Muresan et al. showed similar results, with a higher sensitivity for detecting medial meniscal tears compared to lateral meniscal tears (88.8% versus 70%) [30]. A cadaveric study by Selby et al. correlated the sonographic features and cadaveric findings of meniscal tears and showed that the sonographic visualization of lateral meniscal tears was challenging due to the complex overlying soft tissue structures, particularly the popliteus tendon [31].

Our study also showed that the false negative and false positive rates for the ultrasound detection of meniscal changes were better than the values reported in the literature (13.8% and 15.1%, respectively) [32]. This may be explained by the interval improvement in ultrasound machines, software packages, and image processing, and the musculoskeletal training of the sonographers, since the prior study, which was published in 2008 [32]. These results suggest that there are limitations to ultrasound in detecting specific meniscal pathologies leading to potentially false-negative results. Our study found that the most prevalent patterns of meniscal changes associated with false-negative ultrasound results in detecting medial and lateral meniscal changes were radial tears followed by horizontal tears. Similar results were shown by Akatsu et al. [24]. A study by Selbey et al. showed that radial tears were the most difficult to visualize, particularly if the tear was <5 mm wide [31].

Knee injuries are a common clinical entity and often lead to meniscal tears [4]. Patients with meniscal tears commonly present with a variety of symptoms, such as knee locking, pain, and swelling, and, in chronic cases, could progress to cartilage degeneration and osteoarthritis [2,4,5]. Imaging tests are performed as the standard of care, which includes knee radiographs and MRI [11]. MRI is the gold standard for meniscal tear diagnosis with a sensitivity and specificity of 93% and 88% for medial meniscal tears, and 79% and 96% for lateral meniscal tears, respectively. A study by Ahmadi et al. compared the diagnostic accuracy of MRI and ultrasound and showed that ultrasound accuracy for detecting medial meniscal changes was 89.2% compared to the MRI accuracy of 93% [33]. Another study by Cook et al. compared MRI to US in assessing meniscal changes in acute knee tears and found out that ultrasound had superior specificity and PPV compared to MRI [26].

Ultrasound is a community-based inexpensive imaging test with no contraindications. In this study, the accuracy of US in the detection of medial and lateral meniscal tears was ≥90%. This makes US a valuable diagnostic imaging tool for detecting meniscal tears in a community setting. In North America, a community-based knee ultrasound costs approximately USD 100 compared to USD 400 for a knee MRI scan. Considering that there are approximately 850,000 meniscal injuries that occur per year in the United States [8,9], community-based US for imaging meniscal tears could be, economically, a more viable option in terms of cost savings and better management of limited resources in a constrained healthcare system.

In addition, the present study identified five characteristic meniscal changes which can be identified using US. These include diminutive meniscus, extruded meniscus, parameniscal cysts, displaced meniscal fragments, and meniscal clefts. Meniscal clefts were the most prevalent meniscal changes seen in the medial meniscus, followed by extrusions. Extrusions, however, were the most prevalent change seen in the lateral meniscus followed by clefts. No prior studies have assessed the prevalence of these characteristic meniscal changes in US.

Our study has several limitations. The retrospective design of this study may have led to a selection bias; however, the study design attempted to reduce this bias by having strict inclusion and exclusion criteria. The three-month window between the ultrasound and MRI is another limitation; however, this timeline was chosen in order to optimize the sample size, as choosing a narrower timeline would have reduced the sample size. Another limitation is that community ultrasound scans can vary depending on the level of expertise of the ultrasound technologists, as well as the type of ultrasound machine. In our setting, all ultrasound technologists were musculoskeletal-trained, and all scans were performed using Phillips machines. Lastly, our study used MRI as a reference standard for meniscal tears without surgical correlation. This is because MRI is widely considered the gold standard for meniscal tears, and also that not all patients in our cohort had undergone knee surgery to allow for surgical correlation.

## 5. Conclusions

The results of this study have shown that the community-based ultrasound scanning of the knee is highly accurate in the detection of meniscal pathology when compared with the gold standard (MRI). A community-based knee ultrasound could provide substantial cost savings for the healthcare system by increasing efficiency, optimizing the management of scarce resources, and helping patients with MRI contraindications. In addition, community-based ultrasound would provide convenience and shorter waiting times for symptomatic patients by expediting patient management with care providers such as orthopedic surgery, rheumatology, and physiatry.

## Figures and Tables

**Table 1:** Distribution of meniscal changes seen in the medial and lateral meniscus.

Meniscal Changes in US	Medial Meniscus	Lateral Meniscus
Cyst	8	8
Cleft	41	12
Diminutive	19	3
Displaced Fragment	8	0
Extrusion	40	14

**Table 2:** Medial meniscal tear frequency and percentage seen on US and MRI. 0 = no meniscus tear; 1 = meniscus tear present.

Medial Meniscus Tears US	US Frequency (%)	MRI Frequency (%)
No tear (0)	164 (65.86%)	146 (58.63%)
Tear present (1)	85 (34.14%)	103 (41.37%)

**Table 3:** False-negative and -positive frequencies of ultrasound in detecting medial meniscal changes.

Medial Meniscal Pathology	False-Negative Frequency	False-Positive Frequency
Bucket handle tears	3	
Radial tears	10	2
Horizontal tears	7	
Complex tears	2	
Meniscal Extrusion		2
Total	22	4

**Table 4:** Lateral meniscal tear frequencies and percentages on US and MRI. 0 = no lateral meniscal tears and 1 = lateral meniscal tears.

Lateral Meniscus US	US Frequency (%)	MRI Frequency (%)
No tear (0)	222 (89.16%)	208 (83.53%)
Tear present (1)	27 (10.84%)	41 (16.47%)

**Table 5:** False-negative and -positive frequencies of ultrasound in detecting lateral meniscal changes.

Lateral Meniscal Pathology	False-Negative Frequency	False-Positive Frequency
Horizontal tears	6	
Complex tears	1	
Radial tears	8	
Diminutive meniscus		1
Total	15	1

## Data Availability

The data are available upon request.
